# Endotoxin markers in bronchoalveolar lavage fluid of patients with interstitial lung diseases

**DOI:** 10.1186/2049-6958-7-54

**Published:** 2012-12-22

**Authors:** Bogumiła Szponar, Lennart Larsson, Joanna Domagała-Kulawik

**Affiliations:** 1 Institute of Immunology and Experimental Therapy, Polish Academy of Sciences, Rudolfa Weigla 12, 53-114, Wroclaw, Poland; 2 Lund University, Department of Laboratory Medicine, Section of Medical Microbiology, Sölvegatan 23, 223 62, Lund, Sweden; 3 Warsaw Medical University, Department of Pneumology and Allergology, Banacha 1a, 02-097, Warsaw, Poland

**Keywords:** Bronchoalveolar lavage fluid, Endotoxin, Interstitial lung diseases

## Abstract

**Background:**

Exposure to inhaled endotoxins (lipopolysaccharides, LPS) of Gram-negative bacteria commonly found in indoor environments and assessed in secondary tobacco smoke, has been associated with airway inflammation and asthma exacerbation. The bronchoalveolar lavage fluid (BALf) from patients with interstitial lung diseases (sarcoidosis, lung fibrosis, smoking-related ILD, eosinophilic disorders) was analyzed for the markers of lipopolysaccharide (LPS, endotoxin).

**Methods:**

BALf was obtained from patients with diffuse lung diseases: idiopathic pulmonary fibrosis (n = 42), sarcoidosis (n = 22), smoking-related-ILD (n = 11) and eosinophilic disorders (n = 8). Total cell count and differential cell count were performed. In addition, samples were analyzed for 3-hydroxy fatty acids (3-OHFAs) of 10–18 carbon chain lengths, as markers of LPS, by gas chromatography-tandem mass spectrometry.

**Results:**

The highest LPS concentration was found in patients with eosinophilic disorders and the lowest in patients with sarcoidosis (p< 0.05) followed by the lung fibrosis and the sr-ILD patients. The difference between LPS in BALf with extremely high eosinophil proportion (> 25%) and those with lower proportion was also significant (p = 0.014). A significant correlation was found between LPS and eosinophils, but not between LPS and lymphocytes, neutrophils, or macrophages count.

**Conclusions:**

A positive relationship of LPS and eosinophilic pulmonary disorders may be linked to a persistent eosinophil activation mediated by Th2 pathway: chronic endotoxin exposure would intensify Th2 pathway resulting in fibrosis and, at the same time, eosinophil stimulation, and hence in eosinophilic pulmonary disorders.

## Background

Exposure to inhaled endotoxins (lipopolysaccharides, LPS), components of the cell wall of Gram-negative bacteria which are ubiquitous e.g. in the indoor environment, has been associated with progression of chronic lung diseases and asthma exacerbation [1]. LPS exposure in indoor environments is assessed by chemical markers - 3-hydroxy fatty acids (3-OHFAs), exclusively present in lipid A, the most conservative part of lipopolysaccharide [2-5]. Large amounts of LPS were detected in the mainstream and second hand cigarette tobacco smoke [6,7]. Here we assessed the LPS markers in bronchoalveolar lavage fluid (BALf) from patients suffering from interstitial lung diseases (ILD) regarding possible relationship of endotoxin deposited in alveolar space. Endotoxin could be an etiological agent and biomarker with potential diagnostic application in the ILD.

We studied a relationship between LPS and cell patterns in BALf from patients with smoking-related interstitial lung diseases, including pulmonary fibrosis. Patients with sarcoidosis, all non-smokers, were encompassed as a comparison group. The BALf examination is decisive in smoking-related interstitial lung diseases [8] and in sarcoidosis [9]; in other lung diseases is regarded as a supplementary method. The usefulness of BALf examination as low invasive and well standardized technique in the diagnosis of diffuse lung diseases was widely documented [10-13].

Environmental endotoxin when respired may trigger either the innate or adaptive immunity, depending on concentration and time of exposure [14]. The endotoxin in BALf could therefore reflect a role of air-borne LPS (e.g. from tobacco smoke or organic dust) in initiating the interstitial pulmonary diseases of some clinical manifestations. Indeed, it is widely accepted that environmental factors are important in sarcoidosis pathogenesis, as well as in hypersensitivity pneumonitis [15,16]. In their recent review Seibold and Schwartz [17] identified tobacco smoke, air pollution and LPS as three key factors capable of modifying the genetic susceptibility to lung diseases. Also, in an experimental study on idiopathic pneumonia after allogeneic bone marrow transplantation in mice, LPS was described as the principal agent triggering pulmonary damage by releasing inflammatory cytokines [18].

The role of exposure to inhalable agents, both known (tobacco smoke, allergens) or unidentified, is strongly considered in yet unknown aetiology of interstitial lung disease. We aimed to analyze the relationship of endotoxin present in BALf of patients with different types of well-defined ILD.

## Methods

BALf was obtained from patients with diffuse lung diseases. All patients gave informed consent to bronchoscopy and sampling of BALf and the study was approved by the Ethics Committee at the Medical University of Warsaw (No. KB/106/2004). Eighty three individuals in total were involved in the study, suffering from sarcoidosis (n = 22, mean age 42 yr., range 26–73 yr., 12 male/10 female), smoking-related interstitial lung diseases (sr-ILD) (n = 11, mean age 53 yr., range 27–77 yr., 8 males/3 females), eosinophilic disorders (n = 8, mean age 53 yr., range 30–69, 3 males/5 females), and lung fibrosis (n = 42, mean age 62 yr., range 30–80 yr., 27 males/15 females). Diagnosis of interstitial lung diseases was established according to ATS/ERS International Multidisciplinary Consensus Classification of the Idiopathic Interstitial Pneumonias [19]. The following diseases were included in the group of eosinophilic disorders: chronic eosinophilic pneumonia (CEP), Churg Strauss Syndrome, and Wegener granulomatosis. The large group of lung fibrosis patients included patients with lung fibrosis in the course of hypersensitivity pneumonitis, with connective diseases, and with idiopathic pulmonary fibrosis (IPF).

At the time of collecting the BAL material none of the patients suffered from any known ongoing infection. BALf was collected according to the guidelines of Polish Respiratory Society [13], in line with the international guidelines [20], using 200 mL of saline, and total and differential cell counts were performed. The mean volume of recovery fluid was 100 mL. Cell smears were prepared immediately after the sample had been received at the laboratory, according to the guidelines [13]. After filtration the fluid was centrifuged for 10 min at 400 × g, then the cell pellet was resuspended in PBS. Supernatants were collected in six 5-mL tubes and frozen at −20°C. Total cells were counted using a Bürker chamber whereas differential cell counting was performed on slides stained with the May-Grünwald-Giemsa method. Three hundred cells were used for differential cell counting. The proportion and number of alveolar macrophages (AM), lymphocytes (L), polymorphonuclear granulocytes (PMN) and eosinophils (Eo) were calculated. The smears of all samples were stained with haematoxylin-eosin and interference from any possible malignant cells was excluded. The presence of any abnormal constituents (cells, acellular material, microorganisms) was studied by using light microscopy (magnification × 1000).

BALf supernatants were lyophilized, weighted, and prepared for analysis. In brief, samples were heated in 2 M methanolic HCl at 80°C 18 hours. The liberated fatty acid methyl esters were extracted with heptane and the hydroxylated fatty acid methyl esters were purified by solid phase extraction as previously described [21]. Prior to analysis, the hydroxy fatty acid methyl esters were transformed to trimethylsilyl ethers. Samples thus obtained were kept at 4°C until analysis by gas chromatography-tandem mass spectrometry (GC-MSMS) as described previously [22]. Internal standard was added to quantify the data [23].

Apart from BALf supernatants we also analyzed BALf cell pellets in five samples from patients with different lung diseases.

### Statistical analysis

The Kruskal-Wallis test was applied for data comparisons, since the data were non-normally distributed, and the Spearman rank test was applied for data correlations. Statistical significance was set at p < 0.05.

## Results

No bacteria or other microbes were found in any of the cell smears.

### Total and differential cell counts in BALf

The total cell count was highest in the sr-ILD patients and lowest in the sarcoidosis patients (Table 1). Alveolar macrophages (AM) predominated in all patient groups (particularly in the sr-ILD patients) except in the patients with eosinophilic disorders where eosinophils accounted for as much as 51% of the studied cells. Notably, eosinophils accounted only for 0.6 – 2% of the cells in the other patient groups whereas PMN for 2-10%.

**Table 1:** Total and differential cell count in BALf from patients with interstitial lung diseases

	**TCC [x10**^**6**^**]**	**AM [%]**	**L [%]**	**PMN [%]**	**Eo [%]**
**sarcoidosis n= 22**	19 (12–25)	47 (33–60)	36 (24–56)	6 (4–11)	0.6 (0–2)
**lung fibrosis n**=**42**	29 (18–40)	48 (30–81)	15 (2–33)	10 (5–25)	4 (1–14)
**sr-ILD**^**1**^**n**=**11**	59 (30–68)	93 (90–94)	2 (1–4)	2 (1.5-3)	2 (1–6)
**eosinophilic disorders n**=**8**	22 (13–31)	13.5 (7.5-45)	6 (1.5-8.75)	8 (3.25-17)	51 (37–82)

^1^Smoking-related ILD.

Data expressed as median (p25-p75).

AM, alveolar macrophages; Eo, eosinophils; L, lymphocytes; PMN, polymorphonuclear granulocytes; TCC, Total cell count.

### Lipopolysaccharides in BALf

LPS markers were detected in all BALf samples, both in the supernatants and the pellets. The levels of 3-OH FAs were much higher in the pellets than in the supernatants, with mean values of 536 and 12.1 pmol LPS/mg, respectively.

**Figure 1:**
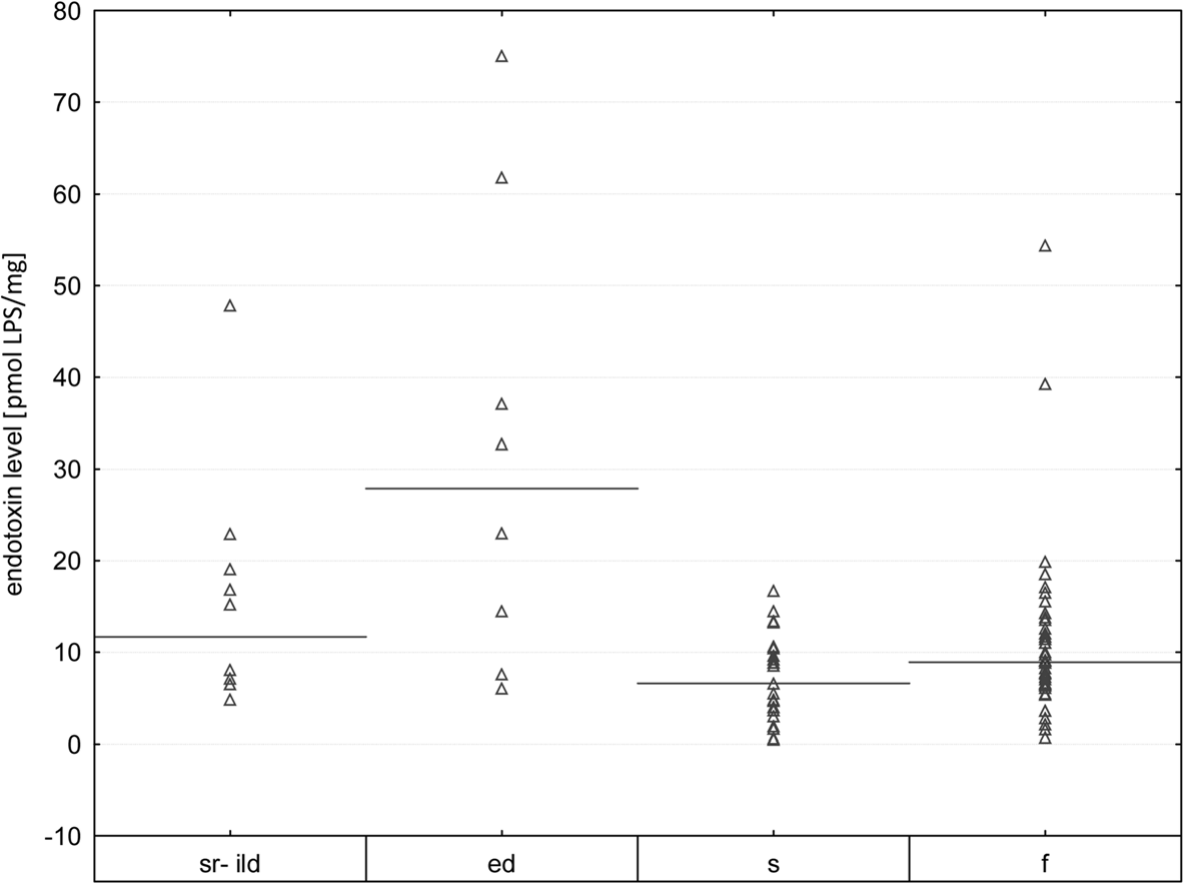
**LPS in BALf supernatants.** Data expressed as median values (lines); ed, eosinophilic disorders; f, fibrosis; s, sarcoidosis; sr-ILD, smoking related interstitial lung diseases.

Patients with sarcoidosis presented the lowest concentrations of LPS in the BALf supernatants followed by the lung fibrosis and the sr-ILD patients. The highest concentrations were found in patients with eosinophilic disorders (Table 2, Figure 1). The differences between levels of LPS in BALf from patients with sarcoidosis and those with eosinophilic disorders were statistically significant (p < 0.05).

**Table 2:** LPS (pmol/mg) in supernatants of BAL from patients with interstitial pulmonary disorders

	**n**	**endotoxin [median]**	**p25**	**p75**
sarcoidosis	22	6.61*	3.68	10.43
lung fibrosis	42	8.91	6.53	13.12
sr-ILD^1^	11	11.68	7.16	19.09
eosinophilic disorders	8	27.88*	11.05	49.45

^1^ smoking related interstitial lung diseases.

*significant difference in Kruscal Wallis test.

P < 0.05.

Concerning the distribution in relation to cell type predominance in BALf samples (lymphocytes higher than normal value 20%, neutrophils higher than 5%, and eosinophils higher than 0.5%), we found a significant increase in LPS in samples with elevated proportions of eosinophils (p = 0.003).

The BALf sample contains cellular and soluble components from distal airways and alveolar space, and the differential inflammatory cell count serves as a basis for the diagnosis. Typical cells are alveolar macrophages, lymphocytes, neutrophils and eosinophils (normal values: > 80%, < 20%, < 5% and < 0.5%, respectively).

**Figure 2:**
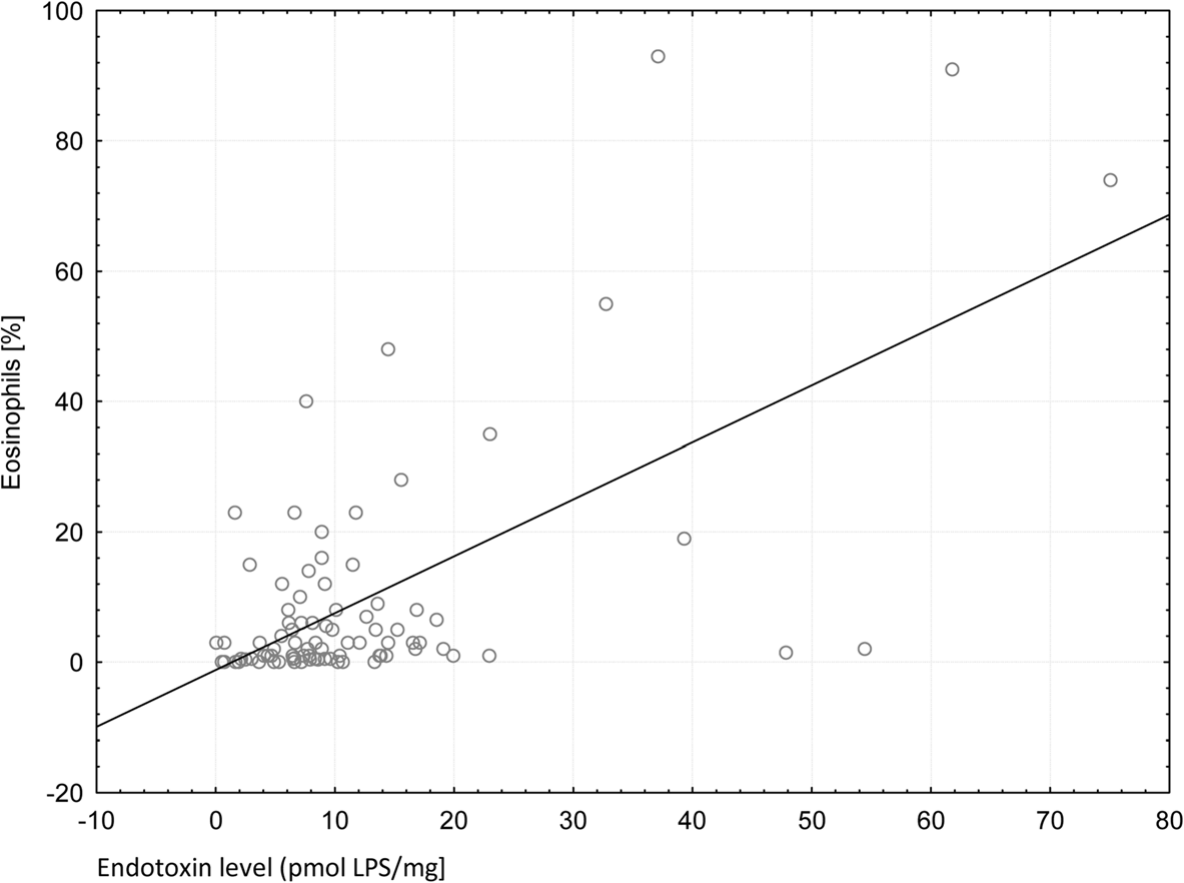
Correlation of LPS with proportion of eosinophils (significant in Spearman test).

The difference between LPS in BALf with extremely high eosinophil proportion (> 25%) and those with lower proportion (< 25%) was also significant (median value 15.6 vs 8.0 pg/ml, p = 0.014). Moreover, a significant correlation of the percentage (R = 0.37, p < 0.05) and the total number (R = 0.35, p < 0.05) of eosinophils with endotoxin concentration was observed (Figure 2).

**Figure 3:**
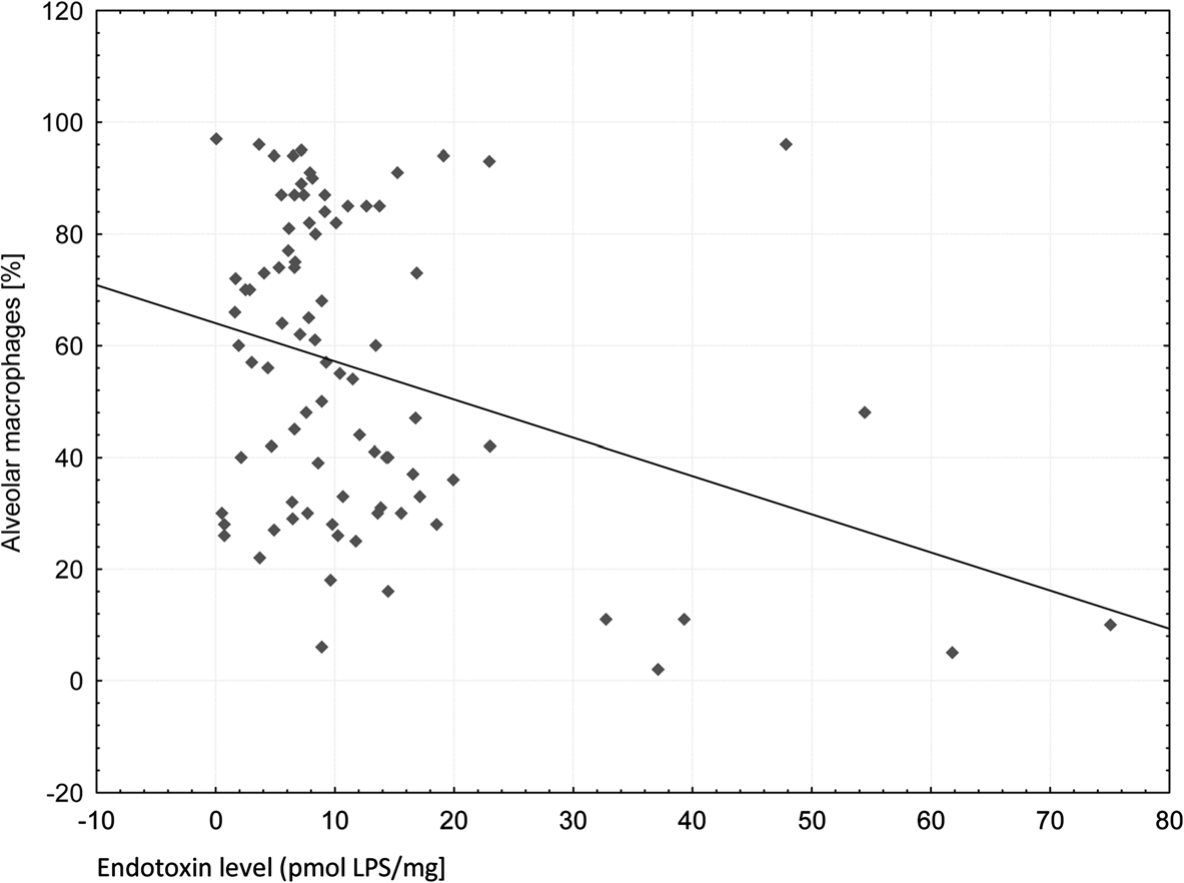
Negative correlation of alveolar macrophages proportion with LPS in BAL (R = −0.25, p < 0.05).

No significant correlation between LPS and lymphocytes, neutrophils, or macrophages count was observed. A significant negative correlation between LPS and the proportion of macrophages was found (R = −0.25, p < 0.05) (Figure 3).

## Discussion

Here we report for the first time the endotoxin load in BALf of patients with different interstitial lung diseases including those involving the cigarette tobacco smoke exposure. Earlier reports on the presence of airborne endotoxin in tobacco smoke [6,24] provided the rationale for the study; notably, LPS is one of the strongest biological triggers of inflammation. The determination of chemical markers of endotoxin has been proven to accurately assess LPS levels in complex biological matrices, and it, for the first time, was here applied in BALf supernatants.

Patients with lung fibrosis, sarcoidosis, smoking-related interstitial lung diseases and eosinophilic disorders were involved in the study, and LPS markers were detected in all BALf samples. Among all patients, sarcoidosis (a disease not associated with tobacco smoking) appeared to be the least related to the level of LPS in BALf, while patients with sr-ILD, a disease associated with smoking, revealedhigherLPS concentrations, and the largest group – the pulmonary fibrosis patients - presented median LPS concentrations in BALf. We found significantly higher BALf LPS concentrations in cases of eosinophilic disorders, demonstrated both as eosinophil proportion and number (Table 2, Figure 1).

Neutrophils, macrophages and lymphocytes are typically the most significant inflammatory cells found in the airways of patients with COPD, while eosinophils may play a role in triggering inflammation in early COPD [25]. The association between LPS and eosinophils found in the present study is important since eosinophils in blood are responsible for activation and perpetuation of allergic inflammatory reactions. The normal proportion of eosinophils in BALf cell smear is 0.5%; eosinophilic disorders are recognized when the proportion is rising as high as >25% [26]. Domagała-Kulawik et al. [27] reported elevated percentages over normal value in 22% of patients with ILD. The mean recorded percentages were 1.9% in sarcoidosis, 3.9% in hypersensitivity pneumonitis, and 4.5% in IPF.

Surprisingly, there was no correlation of LPS with proportion or absolute number of neutrophils, although it has previously been shown that in healthy volunteers LPS inhalation causes a marked increase in circulating neutrophils and a moderate increase in monocytes/macrophages or lymphocytes [28]. Also, an increase in neutrophils, but not in monocytes/macrophages or lymphocytes, was observed in BALf three hours after inhalation [29]. The negative relation of LPS to macrophages (R = −0.25, p < 0.05) in our study may reflect an absence of any current inflammatory reaction with endotoxin involvement, because macrophages are mostly active in the initial recognition of LPS by CD14 receptors, triggering epithelial cells to inflammatory response [30]. In chronic exposure to environmental endotoxin the role of lung macrophages is more complex, i.e. promoting phagocytic activity. In case of long-time exposure to airborne endotoxin (i.e. in a working environment or tobacco smoke) the defending role of macrophages is mostly limited to phagocytosis. It can result in decreased number and proportion of macrophages recorded in BALf in chronic lung diseases, especially in a context of vast eosinophils migration into the lungs.

## Conclusions

Local instillation of LPS to the airways causes an acute increase in inflammatory mediators and cells [14], whereas chronic (e.g. environmental) exposure to endotoxin may affect regulatory system through dendritic cells and T regulatory cells, balancing the Th1>Th2 dependent immune response. Along with this interpretation our results suggest that environmental LPS exposure (i.e. occupational exposure, tobacco smoke and ETS) represents a new, important factor in the pathogenesis of interstitial diseases [1]. Noteworthy, the Th2 pathway strongly promotes lung fibrosis, and sarcoidosis was recently recognized to be a Th1/Th17 disorder [31,32]. As the persistent eosinophil activation is mediated by Th2 pathway, we postulate that prolonged endotoxin exposure exacerbates Th2 pathway resulting in fibrosis and, at the same time, eosinophil stimulation. Such a mechanism would explain the simultaneous increase of endotoxin and eosinophils in BALf.

Studies on the role on prolonged endotoxin exposure in relation to various interstitial lung disorders will be continued, especially regarding the role that LPS plays as a potent stimulator of the inflammatory response and a ligand to Toll-like receptor 4, critical in human innate immunity of respiratory system.

## Abbreviations

3-OHFAs: 3-hydroxy fatty acids; AM: Macrophages; BALf: Bronchoalveolar fluid; CEP: Chronic eosinophilic pneumonia; Eo: Eosinophils; ETS: Environmental tobacco smoke; ILD: Interstitial lung diseases; IPF: Idiopathic pulmonary fibrosis; L: Lymphocytes; LPS: Lipopolysaccharide; PMN: Polymorphonuclear granulocytes; sr-ILD: Smoking-related-ILD.

## Competing interests

Financial/nonfinancial disclosures: The authors have reported no significant conflicts of interest exist with any companies/organizations whose products or services may be discussed in this article.

## Authors' contributions

BS contributed to conception and design of the study, carried out the GCMS analysis of endotoxin markers and drafted the manuscript; LL was involved in interpretation of data and drafting the manuscript; JD-K contributed to conception and design of the study, collected clinical material, performed the statistical analysis and drafted the manuscript. All authors read and approved the final manuscript.
